# Low Prostate Concentration of Lycopene Is Associated with Development of Prostate Cancer in Patients with High-Grade Prostatic Intraepithelial Neoplasia

**DOI:** 10.3390/ijms15011433

**Published:** 2014-01-21

**Authors:** Simone Mariani, Luana Lionetto, Michele Cavallari, Andrea Tubaro, Debora Rasio, Cosimo De Nunzio, Gena M. Hong, Marina Borro, Maurizio Simmaco

**Affiliations:** 1 Division of Urology, Department of Surgery, Sant’Andrea Hospital, Rome 00189, Italy; E-Mails: marianisimone@inwind.it (S.M.); andrea.tubaro@uniroma1.it (A.T.); cosimo.denunzio@gmail.com (C.N.); 2 Urology Department, Fabia Mater Hospital, Rome 00171, Italy; 3 Advanced Molecular Diagnostics Unit, Sant’Andrea Hospital, Rome 00189, Italy; E-Mails: marina.borro@uniroma1.it (M.B.); maurizio.simmaco@uniroma1.it (M.S.); 4 Department of Neurosciences, Mental Health and Sensory Organs–Sapienza University of Rome, Piazzale Aldo Moro, 5, Roma 00185, Italy; E-Mail: cavallari.michele@gmail.com; 5 Department of Clinical and Molecular Medicine, Sapienza University of Rome, Piazzale Aldo Moro, 5, Roma 00185, Italy; E-Mail: deborarasio@gmail.com (D.R.); 6 Center for Infectious Diseases, Boston Medical Center, Boston, MA 02118, USA; E-Mail: ghong@wellesley.edu

**Keywords:** lycopene, prostate cancer, prostatic lycopene, HGPIN

## Abstract

Prostate cancer (PC) is a frequent male malignancy and represents the second most diagnosed cancer in men. Since pre-cancerous lesions, *i.e.*, the high-grade prostatic intraepithelial neoplasia (HGPIN), can be detected years before progression to PC, early diagnosis and chemoprevention are targeted strategies to reduce PC rates. Animal studies have shown that lycopene, a carotenoid contained in tomatoes, is a promising candidate for the chemoprevention of PC. However, its efficacy in humans remains controversial. The present study aimed to investigate the relevance of plasma and prostate concentration of lycopene after a lycopene-enriched diet in patients diagnosed with HGPIN. Thirty-two patients diagnosed with HGPIN were administered a lycopene-enriched diet (20–25 mg/day of lycopene; through 30 g/day of triple concentrated tomato paste) for 6 months. A 6-month follow-up prostate biopsy assessed progression to PC. Patients were classified into three groups according to the histopathological features of the 6-month follow-up biopsy results: prostatitis; HGPIN and PC. PSA and plasma lycopene levels were measured before and after the dietary lycopene supplementation. Prostatic lycopene concentration was only assessed after the supplementation diet. Only prostatic lycopene concentration showed significant differences between the three groups (*p* = 0.03). Prostatic lycopene concentration below a 1 ng/mg threshold was associated with PC at 6-month follow-up biopsy (*p* = 0.003). We observed no overall benefits from a 6-month lycopene supplementation, as the rate of HGPIN progression to PC in our population (9/32, 28%) was similar to rates reported in the literature. Baseline PSA levels also showed no significant changes after a lycopene-enriched diet. Our findings point to prostatic lycopene concentration as a promising biomarker of PC. Further prospective longitudinal studies are needed to assess the prognostic role of prostatic lycopene in PC.

## 1. Introduction

Prostate cancer (PC) is the second most frequently diagnosed cancer in men and the sixth leading cause of death from cancer among men worldwide. Due to the latency of tumor progression and the ability to identify the high-grade prostatic intraepithelial neoplasia (HGPIN) as precursor to PC, early diagnosis and chemoprevention are strategies to reduce PC mortality rates. Systematic screening of the male population, mainly by digital rectal examination (DRE) and dosage of the prostate specific antigen (PSA) in serum has increased the number of early stage PC diagnosis.

Nutritional supplementation and chemoprevention have been targeted strategies to prevent tumor progression. These treatments are essentially based on anti-androgen drugs, which suppress hormone stimulation of the prostate [1]. Supplementation of natural compounds and antioxidants (selenium, vitamin E, lycopene) reduces the elevated level of oxidative stress in the PC cellular microenvironment. The hypothesis that antioxidants may act as protective agents against PC is supported by experimental data, which show the involvement of oxidative stress pathways in the physiopathology of PC [2]. Furthermore, signaling via the androgen receptor itself can be modulated by oxidative stress [3].

Lycopene, a non-provitamin carotenoid found primarily in tomato and its derivatives, is a promising chemopreventive agent with a minimal side-effect profile. It has strong antioxidant power, due to high reactivity towards oxygen and free radicals, but also through induction of antioxidant enzymes, such as superoxide dismutase [4]. Moreover, lycopene has the ability to induce cell cycle arrest at the G1 phase and apoptosis in prostate cancer cell lines [5–8] in a concentration-dependent fashion [9]. Yet the preventive action of dietary supplementation of lycopene in humans is controversial, due to inconsistent results among clinical trials [10]. The present study aimed to assess plasma and prostate concentration of lycopene after a lycopene-enriched diet in order to investigate their relevance to progression to PC in patients diagnosed with HGPIN. While most of the studies in this field have only analyzed plasma lycopene concentrations, this study also assessed prostatic lycopene concentrations and found that low prostatic lycopene levels were associated to PC.

## 2. Results and Discussion

**Figure 1.**
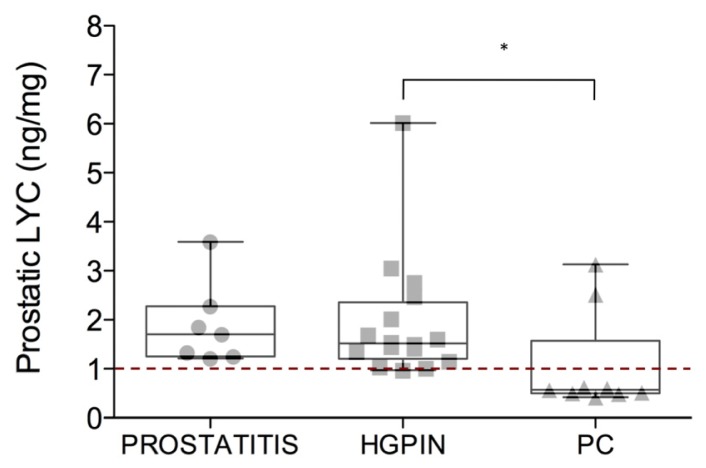
Box plots show group differences in prostatic lycopene concentration. Post hoc comparison analysis (Dunn’s test) showed a significant difference between HGPIN and PC groups. The red dotted line indicates the cut-off value of prostatic lycopene, which corresponds to the lowest quartile of the distribution (1 ng/mg). The line inside each box represents the median, the whiskers include the minimum and maximum of all of the data. Abbreviations: LYC, lycopene; HGPIN, high-grade prostatic intraepithelial neoplasia; PC, prostate cancer. * *p* ≤ 0.05.

After 6 months of lycopene-enriched diet, the 32 HGPIN patients were classified in three groups according to the follow-up biopsy: prostatitis (*n* = 7; 21.8%), HGPIN (*n* = 16; 50.0%) and PC (*n* = 9; 28.2%). Group differences in PSA, plasmatic and prostatic lycopene concentrations before and after lycopene supplementation are summarized in Table 1. Among these variables, only prostatic lycopene concentration showed significant differences between the three groups (Kruskal-Wallis, *p* = 0.03). Post-hoc analysis (Dunn’s test) revealed significant difference between the HGPIN and PC groups only, with prostatic lycopene lower in the PC group (Figure 1).

We found a cross-sectional association between prostatic lycopene concentration below a 1 ng/mg threshold and PC at 6-month follow-up biopsy (Chi-squared test, *p* = 0.003; Table 2). The 1 ng/mg cut-off value of prostate lycopene concentration corresponding to the lowest quartile of the distribution identifies PC with a 95.6% specificity and a 77.8% sensitivity, with a positive predictive value (PPV) of 87.5% and a negative predictive value (NPV) of 91.7% (Table 2). Only one subject with prostate lycopene concentration below this threshold had HGPIN diagnosis at 6-month follow-up biopsy (Table 2; Figure 1). Remarkably, this subject was histologically diagnosed with PC at the 12-month follow-up. None of the subjects with prostate lycopene concentrations above 1 ng/mg developed PC at 1-year follow-up. Both repeated measures and cross-sectional analyses of PSA and plasmatic lycopene levels showed no significant differences between the groups (Friedman and Kruskal-Wallis tests; Table 1).

Taken together, these findings point to prostatic lycopene concentration as a promising biomarker of PC. To our knowledge, only two studies of dietary supplementation of lycopene in patients with prostate cancer have quantified the prostate lycopene concentration [11,12]. The authors reported that the increase in prostatic lycopene levels was accompanied by a decrease in PSA levels, as well as other cancer biomarkers (e.g., oxidative cell damage and apoptotic cell death), which suggested a beneficial effect of lycopene accumulation in the prostate. Our finding that prostatic lycopene was lower in patients with PC compared to those with HGPIN at 6-month follow-up, supports the beneficial role of prostatic accumulation of lycopene. The lack of measurement of prostatic lycopene concentration before lycopene supplementation in our study design did not allow us to assess the dynamics of prostatic lycopene adsorption relative to dietary intake. Although our results suggest that prostatic rather than systemic absorption of lycopene may be a determinant to the development of PC, further studies are warranted to confirm the relevance of prostatic lycopene concentration to PC.

Although several studies investigated lycopene as a prostate cancer preventive agent, no definitive results about its efficacy have been reported [13,14]. In this study we observed no overall benefits from a 6-month lycopene supplementation, since the rate of HGPIN progression to PC in our population (9/32, 28%) was similar to the rates reported in the literature [15]. In addition, we observed no significant changes in PSA in the whole population, as well as in each group separately, after lycopene-enriched diet (Wilcoxon signed rank test).

Most of the studies that have investigated the potential beneficial effect of lycopene in PC prevention only assessed plasmatic lycopene concentrations as a correlate of systemic absorption of lycopene. Some of these studies have found a negative correlation between lycopene levels in plasma and prostate cancer [16–18]. Similarly, baseline plasmatic lycopene concentrations in our study were highest in patients who were diagnosed with prostatitis and lowest in those diagnosed with PC at the 6-month follow-up (Table 1). The difference in plasmatic lycopene concentration among the groups did not reach the threshold for statistical significance, likely due to the small sample size. Yet this result supports the notion of an inverse correlation between plasmatic lycopene levels and prostate cancer [16–18]. It is worth mentioning that baseline plasmatic lycopene concentrations in our study population were higher compared to those commonly reported in the literature (range: 0.13–2.71 μmol/L *vs.* 0.18–0.53 μmol/L) [19,20], likely due to a Mediterranean diet, which implies a relatively higher consumption of tomato. Plasma lycopene levels showed no increase after the 6-month supplementation diet, probably because of a plateau effect. Interestingly, group analysis showed that those patients who developed PC had lower baseline plasmatic levels of lycopene compared to the prostatitis and the HGPIN groups (58% on average lower than overall mean concentration), even though the difference between the groups did not reach statistical significance. After the lycopene supplementation diet, plasma lycopene significantly increased in the PC group (*p* = 0.04). Remarkably, only plasmatic lycopene levels increased following lycopene supplementation, while prostatic lycopene remained low in subjects with PC. These findings further support the relevance of prostate lycopene concentration to PC.

We acknowledge two main limitations of the present study. First, the small sample size limits the general applicability of our findings. Second, the lack of the measurement of prostate lycopene concentration at baseline did not allow us to assess the dynamics of lycopene accumulation in prostate tissue following dietary supplementation and therefore to investigate the association between prostatic lycopene and PC development. Low prostatic lycopene concentration appeared to be a correlate of PC in our study, yet further longitudinal studies are warranted to assess the ability of prostate lycopene concentration to predict HGPIN progression to PC.

## 3. Experimental Section

### 3.1. Patients and Study Design

Thirty-two patients (mean ± SD age = 66.2 ± 6.5) diagnosed with HGPIN were consecutively enrolled from November 2009 through March 2011. The patients were referred to the study through the Urology Department of the Sant’Andrea Hospital in Rome, Italy. The baseline diagnosis of HGPIN was conducted by 12 core transrectal ultrasound-guided prostatic biopsy.

All enrolled patients were instructed to ingest 20–25 mg/day of lycopene through dietary supplementation for six months. Each patient was provided with triple concentrated tomato paste (Mutti S.P.A., Parma, Italy, http://www.mutti-parma.com) and instructed to ingest 30 g/day with one third of a spoon of extra-virgin olive oil in order to enhance the bioavailability of lycopene [21].

Dietary supplementation was self-administered and compliance was monitored by review of the patients’ self-recorded dietary diary. Only subjects consuming 30 g/day of triple concentrate tomato paste for at least 5 days/week were included in the analysis. Two patients were excluded due to non-compliance.

Serum and plasma samples were collected before and after lycopene dietary supplementation to analyze the concentration of PSA and lycopene, respectively. Samples that were not immediately analyzed were stored at −80 °C. After 6 months of lycopene supplementation, a follow-up 14–16 core transrectal prostate biopsy was performed. The patients were categorized to the following three groups according to the follow-up biopsy results: prostatitis, HGPIN or PC. The prostatic lycopene concentration was measured by a biopsy fragment that was stored at −80 °C. According to good clinical practice, all patients were clinically evaluated at a 12-month follow-up.

The study was approved by the Institutional Review Board of Sant’Andrea Hospital and all procedures were conducted in accordance with the Declaration of Helsinki.

### 3.2. Determination of PSA and Plasma Lycopene

Serum PSA was measured according to standard laboratory procedure. Plasma concentrations of lycopene were quantified using a HPLC system coupled with a nano-UV detector (Knauer, Berlin, Germany). Chromatographic separation was carried out on a reverse phase Jupiter 4 μm Proteo (90 Å) column (250 × 1.0 mm) held at 50 °C with a flow rate of 0.08 mL/min. Solvent A was 50% methanol and solvent B was methanol/isopropanol (60/40 *v*/*v*). Elution was performed according to the following gradient: 0–9 min from 80% to 89% B; 9–20 min from 89% to 95% B; 20–21 min from 95% to 80% B; 21–25 min 80% B. Elution of α-tocopherol acetate was used as an internal standard and monitored at 292 nm, whereas lycopene elution was monitored at 473 nm.

### 3.3. Determination of Prostate Lycopene

The prostatic tissue (10–15 mg) was added to 500 μL of milliQ H_2_O (Millipore, Billerica, MA, USA) and homogenized at 20,000 rpm by an ULTRATURRAX T25 (IKA, Staufen, Germany). After the addition of 50 μL of α-tocopherol acetate (2 mg/dL in methanol), the mixture was vortexed and subsequently added with 1 mL hexane and extensively vortexed. After centrifugation (4000 rpm, 10 min), 800 μL of the organic phase was transferred in sterile tubes and dried by vacuum centrifugation. The pellet was vortexed, suspended in 100 μL of methanol and injected into the chromatographic column. The HPLC separation was performed according to the protocol described above.

### 3.4. Statistical Analysis

Statistical analyses were performed using GraphPad/Prism (version 5.0, GraphPad Software Inc., San Diego, CA, USA). Group differences in PSA levels and plasma concentrations of lycopene before and after lycopene dietary supplementation were assessed by Friedman’s test, with time being the repeated measure factor. Group differences in PSA, plasmatic and prostatic lycopene within a single time point were assessed by Kruskal-Wallis test, and Dunn’s test for post hoc analysis. Variables that showed significant differences among the groups were used to subdivide the study subjects according to quartile distribution. Association between quartiles and diagnoses was evaluated using Chi-squared test. Contingency table analysis was carried out to measure sensitivity, specificity, positive and negative predicting values. Wilcoxon signed rank test was used to compare baseline and follow-up levels of PSA and plasmatic lycopene. The significance threshold for statistical testing was ≤0.05.

## 4. Conclusions

Our study found that low prostate lycopene concentrations were associated with PC. Further studies are needed to confirm our results and to investigate the predictive role of prostate lycopene concentrations in patients at risk for PC.

## Figures and Tables

**Table 1.** Group differences in age, PSA, plasmatic and prostatic lycopene concentrations before and after lycopene supplementation.

	All Subjects (*n* = 32)	Prostatitis (*n* = 7)	HGPIN (*n* = 16)	Prostate Cancer (*n* = 9)	*p*
**Age (years)**	66.22 ± 6.53	66.57 ± 7.50	64.44 ± 6.67	69.50 ± 4.41	0.37
**PSA (ng/dL)**					
Pre	8.40 ± 4.85	10.26 ± 7.68	7.51 ± 2.50	8.43 ± 5.36	0.99
Post	7.93 ± 4.77	9.6 ± 6.3	6.87 ± 2.6	8.42 ± 5.67	0.86
**Plasmatic Lycopene (μmol/L)**					
Pre	1.17 ± 0.92	1.42 ± 1.29	1.27 ± 0.89	0.68 ± 0.31	0.06
Post	1.38 ± 0.57	1.23 ± 0.50	1.52 ± 0.65	1.26 ± 0.46	0.58
**Prostatic Lycopene (ng/mg)**					
Post	1.67 ± 1.15	1.89 ± 0.84	1.94 ± 1.25	1.04 ± 1.02	0.03

Data are expressed as mean ± SD. Pre and post are relative to 6-month lycopene-enriched diet. Group comparison: *p*-values (Kruskal-Wallis test) indicate level of significance among the three groups (prostatitis, HGPIN, prostate cancer). Abbreviations: PSA, prostate-specific antigen; HGPIN, high-grade prostatic intraepithelial neoplasia.

**Table 2.** Contingency table showing the relationship between diagnosis and low prostatic lycopene levels according to 1 ng/mg cut-off value, which corresponds to the lowest quartile of the distribution.

	Prostatitis	HGPIN	Prostate Cancer	Total
**Prostatic Lycopene < 1 ng/mg**	0 (0%)	1 (6.3%)	7 (77.8%)	8
**Prostatic Lycopene > 1 ng/mg**	7 (100%)	15 (93.7%)	2 (22.2%)	24
**Total**	7	16	9	32
**Chi-Squared** ***p*-value**	0.003			
**Sensitivity**	77.8%			
**Specificity**	95.6%			
**Positive Predictive Value**	87.5%			
**Negative Predictive Value**	91.7%			

Frequencies are expressed as *n* (%);

HGPIN, high-grade prostatic intraepithelial neoplasia.
